# Draft Genome Sequences of Three *Arcobacter* Strains of Pig and Dairy Cattle Manure Origin

**DOI:** 10.1128/genomeA.00377-14

**Published:** 2014-05-15

**Authors:** Zaky Adam, Kerri Whiteduck-Leveillee, Michel Cloutier, James T. Tambong, Wen Chen, Christopher T. Lewis, C. André Lévesque, Edward Topp, David R. Lapen, Guylaine Talbot, Izhar U. H. Khan

**Affiliations:** a Eastern Cereal and Oilseed Research Centre (ECORC), Agriculture and Agri-Food Canada, Ottawa, Ontario, Canada; b Southern Crop Protection and Food Research Centre, Agriculture and Agri-Food Canada, London, Ontario, Canada; c Dairy and Swine Research Development Centre, Agriculture and Agri-Food Canada, Sherbrooke, Quebec, Canada

## Abstract

The genus *Arcobacter* has been associated with human illness and fecal contamination by humans and animals. Here, we announce the draft genome sequences of three strains of *Arcobacter* species cultured from pig and dairy cattle manure tanks. This information will assist in the characterization of features related to host specificities and identify potential pathogenic health risks to humans and animals.

## GENOME ANNOUNCEMENT

*Arcobacter* species have been implicated in human infections, which has led to these microbes being characterized as potential food-borne and water-borne pathogens (1). Moreover, the presence of *Arcobacter* spp. in livestock indicates that animals may also serve as possible reservoirs for *Arcobacter* species (2). Therefore, the prevalence, relative abundance, sources, and route(s) of human and animal exposures to *Arcobacter* spp. need to be identified and considered health concerns.

In 2010, *Arcobacter* strains AF1430 and AF1440 were recovered from a pig manure tank, and strain AF1581 was isolated from a dairy cattle manure tank from farms located in the southeast region of Ottawa, Ontario, Canada. The genome sequencing project was initiated based on multiple pairwise alignment of 16S rRNA gene, where these *Arcobacter* strains showed high phylogenetic relatedness (91.3 to 97.5% identity) to other known *Arcobacter* species. The whole-genome sequencing of the purified genomic DNA was performed using paired-end sequencing on Illumina HiSeq 2500 with TruSeq version 3 chemistry at the National Research Council Canada (Saskatoon, Saskatchewan, Canada). Following fragmentation, end reparation, and sample tagging, the sequencer produced 101-bp paired-end reads that were obtained from 300-bp inserts, yielding average coverages of 572×, 419×, and 893× for strains AF1430, AF1440, and AF1581, respectively. The program FastQC (http://www.bioinformatics.babraham.ac.uk/projects/fastqc/) was applied to check the quality of the reads. *De novo* assembly was performed using ABySS version 1.3.6 (3). SSPACE version 2.0 (4) was applied to extend and merge the resulting scaffolds based on read-pair information and short overlaps to reduce the number of scaffolds. The gaps between the short scaffolds, which are contained within the large scaffolds, were closed using GapFiller version 1.11 (5) by replacing the unknown nucleotides (Ns) with true nucleotides. The resulting scaffolds of the three *Arcobacter* strains were ordered by alignment to the reference genomes of *Arcobacter butzleri* ED-1 and *Arcobacter cibarius* LMG 21996 using Mauve Contig Mover version 2.3.1 (6–8). The genome G+C contents were relatively low (26.4 to 26.8%) but were close to those of the reference genomes (~27%) (7, 8). The genome information data for each strain are summarized in Table 1.

**TABLE 1:** Summary of data for newly sequenced genomes of three strains of *Arcobacter* species

*Arcobacter* sp. strain	Source	Accession no.	Genome size (bp)	*N*_50_ (bp)	No. of scaffolds (>300 bp)	G+C content (%)
AF1430	Pig manure	JATO00000000	2,236,207	338,260	27	26.41
AF1440	Pig manure	JARU00000000	2,287,768	466,048	29	26.66
AF1581	Dairy cattle manure	JARV00000000	2,260,089	414,791	22	26.84

The draft genomes were further annotated for gene prediction using the RAST annotation server (9). We identified similar, but not identical, numbers of protein-coding sequences in all three strains; strain AF1430 contains 2,223 predicted protein-coding sequences, including 1,636 functional, 241 proposed functional, and 346 hypothetical proteins with 42 predicted noncoding RNAs (39 tRNAs, 1 pseudo-tRNAs, and 2 rRNAs consisting of 1 copy each of 16S rRNA and 23S rRNA genes), compared to strain AF1440, which contains 2,245 predicted protein-coding sequences, including 1,655 functional, 241 proposed functional, and 349 hypothetical proteins with 64 predicted noncoding RNAs (51 tRNAs, 1 pseudo-tRNAs, and 12 rRNAs consisting of 6 copies each of 16S rRNA and 23S rRNA genes). On the other hand, strain AF1581 contains 2,224 predicted protein-coding sequences, including 1,625 functional, 223 proposed functional, and 376 hypothetical proteins with 75 predicted noncoding RNAs (55 tRNAs, 1 pseudo-tRNAs, and 19 rRNAs consisting of 10 copies of 16S rRNA and 9 copies of 23S rRNA genes).

### Nucleotide sequence accession numbers.

The draft genome sequences of the *Arcobacter* strains AF1430, AF1440, and AF1581 in this study have been deposited as whole-genome shotgun projects at DDBJ/EMBL/GenBank under the accession no. JATO00000000, JARU00000000, and JARV00000000, respectively. The version of each strain described in this paper is the first version, with accession no. JATO01000000, JARU01000000, and JARV01000000.
